# Whole Genome PCR Scanning Reveals the Syntenic Genome Structure of Toxigenic *Vibrio cholerae* Strains in the O1/O139 Population

**DOI:** 10.1371/journal.pone.0024267

**Published:** 2011-08-31

**Authors:** Bo Pang, Xiao Zheng, Baowei Diao, Zhigang Cui, Haijian Zhou, Shouyi Gao, Biao Kan

**Affiliations:** National Institute for Communicable Disease Control and Prevention, Chinese Center for Disease Control and Prevention, State Key Laboratory for Infectious Disease Prevention and Control, Changping, Beijing, China; Charité-University Medicine Berlin, Germany

## Abstract

Vibrio cholerae is commonly found in estuarine water systems. Toxigenic O1 and O139 V. cholerae strains have caused cholera epidemics and pandemics, whereas the nontoxigenic strains within these serogroups only occasionally lead to disease. To understand the differences in the genome and clonality between the toxigenic and nontoxigenic strains of V. cholerae serogroups O1 and O139, we employed a whole genome PCR scanning (WGPScanning) method, an rrn operon-mediated fragment rearrangement analysis and comparative genomic hybridization (CGH) to analyze the genome structure of different strains. WGPScanning in conjunction with CGH revealed that the genomic contents of the toxigenic strains were conservative, except for a few indels located mainly in mobile elements. Minor nucleotide variation in orthologous genes appeared to be the major difference between the toxigenic strains. rrn operon-mediated rearrangements were infrequent in El Tor toxigenic strains tested using I-CeuI digested pulsed-field gel electrophoresis (PFGE) analysis and PCR analysis based on flanking sequence of rrn operons. Using these methods, we found that the genomic structures of toxigenic El Tor and O139 strains were syntenic. The nontoxigenic strains exhibited more extensive sequence variations, but toxin coregulated pilus positive (TCP+) strains had a similar structure. TCP+ nontoxigenic strains could be subdivided into multiple lineages according to the TCP type, suggesting the existence of complex intermediates in the evolution of toxigenic strains. The data indicate that toxigenic O1 El Tor and O139 strains were derived from a single lineage of intermediates from complex clones in the environment. The nontoxigenic strains with non-El Tor type TCP may yet evolve into new epidemic clones after attaining toxigenic attributes.

## Introduction

*Vibrio cholerae* is autochthonous to warm aquatic ecosystems [1]. More than 200 O-antigen serogroups of *V. cholerae* have been identified; however, only serogroups O1 and O139 strains have been associated with cholera epidemics and pandemics [2]. Of the seven cholera pandemics, the sixth and seventh were caused by O1 classical and El Tor biotype *V. cholerae*, respectively. Epidemics of the O139 cholera emerged in 1992 in Bangladesh and India [3], [4]. This group currently remains confined to Asia.

The cholera toxin gene *ctxAB* is located in the lysogenic bacteriophage, CTXΦ, on the *Vibrio* genome [2]. *ctxAB* can be transferred between toxigenic strains and nontoxigenic strains by lysogenic infection with CTXΦ, which is facilitated by the receptor, toxin coregulated pilus (TCP) [5]. Therefore, CTXΦ plays an important role in the emergence of new epidemic clones of *V. cholerae*. Several variants of TCP exist. Each variant of TCP can mediate infection of certain nontoxigenic strains by different types of CTXΦ at varying efficiency [6].

Different clones of *V. cholerae* are continuously being isolated from environmental water and the human population. The toxigenic strains, especially those responsible for epidemics, are highly clonal; however, the nontoxigenic strains exhibit obvious genetic diversity [2], [7], [8], [9]. Although the overall gene content of *V. cholerae* has been defined, the gene arrangements and presence of insertion and/or deletions (so-called indels) in the genomes of *V. cholerae* remain to be defined. Indeed, a recent publication described a comparative analysis of the whole genome sequences by which shift and drift transition events of cholera pandemic clones were defined [9].

Whole genome PCR Scanning (WGPScanning) is a systematic PCR analysis, which can provide insight about gene arrangement and fragment insertion/deletion in multiple strains of bacteria with highly similar genetic backgrounds [10]. In this method, a series of primer pairs are designed based on the sequence of the reference strain to cover the whole genome. The adjacent amplicons overlapped at each end. These primer pairs are then used to amplify the genomes of the test strains. This technology has been successfully applied to *Escherichia coli* O157:H7 [10], [11], *Staphylococcus aureus* [12], and *Streptococcus* [13]. These studies have revealed an unexpectedly high level of genomic plasticity in bacteria.

Large chromosomal fragment rearrangement is a common form of genome variation. Other than rearrangements caused by bacteriophage and phage-like elements [10], an *rrn* operon recombination event can mediate large DNA fragment rearrangement [14]. The PFGE profile of I-*Ceu*I digested genomic DNA from various bacteria has been used in a *rrn* operon-related analysis [15]. The diversity of ribotypes in El Tor has been suggested to be a result of recombination between different *rrn* operons [16]. However, whether large chromosomal fragment rearrangements in the El Tor genome can be mediated by recombination between different *rrn* operons has not been determined.

The genomic organization of both toxigenic and nontoxigenic O1 and O139 *V. cholerae* has been investigated by comparative genome hybridization (CGH) [17], [18]. However, due to the asymmetric nature of the gene chip, gene arrangements and fragment insertions compared to the reference strain remain unidentifiable. We then performed WGPScanning with O1 and O139 strains which had been analyzed in the CGH test. By combining the results from CGH and WGPScanning, we hypothesize that the genome structure of toxigenic *V. cholerae* O1 and O139 to be syntenic. *rrn* operon-mediated large chromosomal fragment rearrangement was not observed among the strains studied. Compared with the toxigenic strains, the nontoxigenic strains appear to have experienced more genetic recombination events and exhibit more extensive sequence variation.

## Results

### Overview of WGPScanning

**Figure 1:**
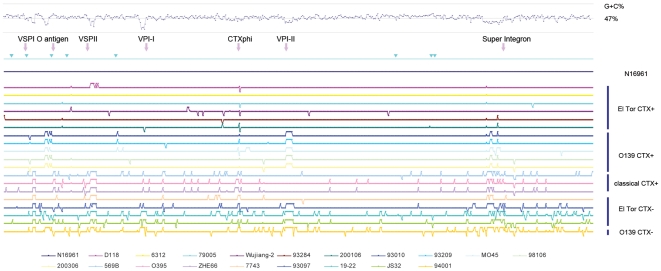
Amplification results for the tested strains compared with N16961. For each of the tested strains, the final results of the amplification for each primer pair were assigned values of 1, 2 or 0.75 when the amplicons were identical to, larger than or smaller than those of N16961, respectively. For the region that did not produce any amplicons, the value of 0 was assigned. Data from the 2 chromosomes were combined, and the amplification results were displayed according to the order they were in N16961. A “1” was assigned to all of the amplicons in N16961; therefore, the results for N16961 are shown as one straight line in Microsoft Excel 2003. Nineteen curves were produced for the 19 tested strains with Microsoft Excel 2003. Finally, the 19 curves were integrated into one figure. To ease comparison, the figure was flipped horizontally. The upward spikes represent no amplicon or that the amplicons in the tested strains were smaller than those in the reference strain. The downward spikes represent amplicons in the tested strains were larger than those in the reference strain. The GC content curve is displayed at the top. The mobile elements, the *rrn* operons and the I-*Ceu*I site in N16961 are annotated according to their position in the genome. The blue inverted triangle displayed on the N16961 genome represents the position of *rrn* operons.

**Figure 2:**
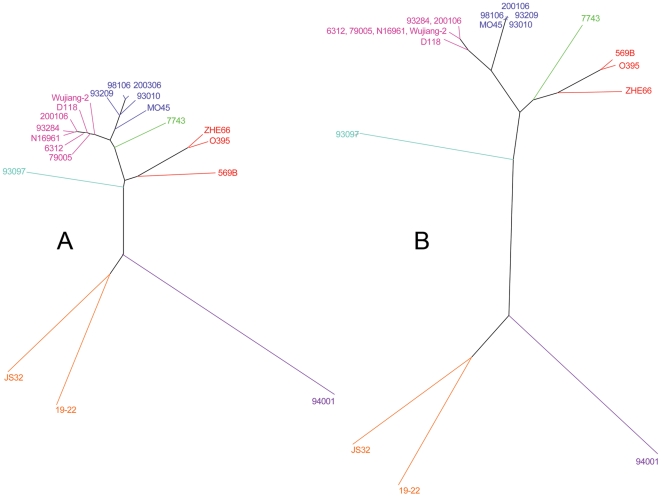
Comparison of cladograms based on WGPScanning (A) and CGH (B) data for the tested strains. The UPGMA trees shown were calculated from difference matrices with BioNumerics. In WGPScanning analysis, amplicons in the tested strains that were identical to, larger than or smaller than those of N16961 were assigned values of 1, 2 or 0.75, respectively. For the region that did not produce any amplicons, a value of 0 was assigned. In CGH analysis, final absent or present calls for each gene of each tested strain were assigned the value 0 or 1, respectively.

All primer pairs yielded amplicons of the expected sizes from the template, N16961. The 11,440 PCR reactions performed on the 19 tested strains are summarized in Figure 1 and Table 2. A cladogram was reconstructed based on the WGPScanning results (Figure 2A). The similarity coefficient between cladograms was determined to be 91.9%, based on the results of the WGPScanning and CGH (Figure 2B) for the 20 strains.

The initial PCR scanning of the 19 tested strains yielded 10,243 PCR products from a total of 10,868 PCR reactions, indicating an amplification success rate of 94.2%. Among these amplicons, 9,627 (88.6%) were indistinguishable in size from those of N16961; 165 exhibited appreciable size differences (89 were larger than the amplicons from N16961, and 86 were smaller). This suggests that an insertion or deletion event had occurred. The remaining 625 fragments could not be amplified. The extent of the differences in the amplification results varied from strain to strain (Table 2). In general, the average difference between the amplification results for the nontoxigenic strains was high, while the difference for toxigenic strains was low. For the nontoxigenic El Tor and O139 strains, the average difference between the amplification results (including fragment increase, decrease and non-amplifiable status) was 23.8% and 13.0%, respectively; toxigenic El Tor and O139 strains presented significantly lower values: 4.6% and 2.2%, respectively (Table 2). The maximum amplification difference was observed in nontoxigenic O139 strain 94001 (23.8%, 136/572). The minimum amplification difference was observed in toxigenic El Tor strain 9328 (1.4%, 8/572). These differences were found to be clustered in mobile regions within the genome (Figure 1).

### Genome content and gene arrangement of toxigenic El Tor and O139 strains

The initial PCR scanning of six toxigenic El Tor tested strains (not including N16961) resulted in 3,386 PCR amplicons from 3,432 PCR reactions (98.7% success rate); the five toxigenic O139 strains resulted in 2,754 amplicons from 2,860 reactions (96.3% success rate). The rates of successful amplification for each of the six toxigenic El Tor strains ranged from 97.9% to 99.1%. Each of the five O139 strains generated success rates ranging from 94.9% to 97.6%.

Of the amplicons produced for each group, 3,358 (97.6%) from the El Tor strains and 2,724 (95.2%) from the 0139 strains were indistinguishable in size from those produced from N16961. Twenty-eight of the El Tor amplicons differed in size: 13 were larger than those from N16961, and 15 were smaller. Similarly, Twenty-five of the O139 amplicons differed in size: 12 were larger than those from N16961, and 13 were smaller.

No amplicons were obtained from the remaining 46 El Tor and 106 O139 fragments. The fragments that presented different amplification results were found to be randomly distributed on either one of the two chromosomes.

Compared with N16961, the maximum difference between results from the toxigenic El Tor and O139 strains were 1.4% and 2.1%, respectively (Table 2). The variation of CGH results from these strains was less than 1% [18]. By comparing the WGPScanning results to those from the CGH analysis, we found that the vast majority of the results from these two distinct approaches were consistent with one another (Figure S1 and Figure S2). For example, if certain open reading frames (ORFs) were not amplified in WGPScanning, those ORFs were also not detected by CGH, and vice versa. However, this was not true for 31 of the toxigenic El Tor fragments and 24 of the O139 fragments. For these fragments, CGH results were positive, and the WGPScanning results were negative.

To address the discrepancy between these two results, we redesigned the corresponding primers and repeated the WGPScanning. As a result, the vast majority of those inconsistent fragments (26/31 of the El Tor and 21/24 of the O139) yielded the same amplicons as those of the reference strain. Therefore, the results from WGPScanning were consistent with results from CGH. This suggested that minor nucleotide variation might exist in the corresponding ORFs and might be responsible for the observed differences in amplification.

### Genome content and gene arrangement of non-toxigenic O1 El Tor and O139 strains

In general, the nontoxigenic El Tor and O139 strains exhibited greater diversity than did the toxigenic strains, as determined by WGPScanning. In CGH test, the TCP^+^ nontoxigenic El Tor strains were found to be more similar in genome content to the toxigenic El Tor stains than they were to the TCP^−^ nontoxigenic strains [18]. A similar result was found using WGPScanning. Identical results were got in the region coding for O antigen (VC0241–VC0254, VC0259–VC0263) in all of the tested O1 strains in both CGH test and WGPScanning analysis.

#### (i) Nontoxigenic El Tor strains

Two TCP**^+^** nontoxigenic El Tor strains (93097 and 7743) and two TCP^−^ nontoxigenic El Tor strains (19–22 and JS32) were included in this study. In 1,144 reactions, 1,067 and 986 amplicons were obtained in the two TCP^+^ and TCP^−^ nontoxigenic El Tor strains respectively. The successful amplification rates were 93.3% and 86.2% in the TCP**^+^** and TCP**^−^** nontoxigenic El Tor strains respectively. Among the 1,067 and 986 amplicons, 1,053 (TCP**^+^** nontoxigenic El Tor strains, 98.7%) and 937 (TCP**^−^** nontoxigenic El Tor strains, 95.0%) amplicons were indistinguishable in size from those of N16961. Fourteen and 49 amplicons in TCP**^+^** and TCP**^−^** nontoxigenic El Tor strains respectively displayed differences in size. No amplicons were obtained from the remaining 77 and 158 fragments in TCP**^+^** and TCP**^−^** nontoxigenic El Tor strains respectively (Table 2).

In the majority region of the nontoxigenic strain genomes, the WGPScanning and CGH methods gave consistent results. That is to say, for certain genome region in a tested strain, if an amplicon identical to that in N16961 was got in WGPScanning the same region could also be detected by CGH (Figure S1 and Figure S2). A rate of consistency was used to evaluate the consistency between results of these two methods. The rate of consistency was calculated as N/572 (N, the number of amplicons that give consistent result between these two method; 572, the number of PCR in one strain). For TCP^+^ nontoxigenic strains, the average rate of consistency was 96.0% (549/572); for TCP^−^ nontoxigenic strains the rate was 89.2% (510/572). The TCP^+^ nontoxigenic strain 7743 (El Tor type *tcpA*) had a similar consistency rate to that of toxigenic El Tor strains. Another TCP^+^ nontoxigenic strain, 93097 (*tcpA* variant), had a consistency rate of 94.1%. This was lower than that of 7,743 (98.1%) but higher than the highest rate observed for the TCP^−^ nontoxigenic strain JS32 (90.7%, 519/572). Moreover, the average number of amplicons showing different size from TCP^−^ nontoxigenic strains was more than two-fold higher than that from TCP^+^ nontoxigenic strains (104 *vs.* 46), suggesting that more insertion or deletion existed in the TCP^−^ nontoxigenic strains.

#### (ii) Nontoxigenic O139 strains

Four hundred seventy-three amplicons were obtained from 572 reactions carried out with nontoxigenic O139 strain 94001 (82.7%, 473/572). Among the 473 amplicons, 436 (92.2%) were undistinguishable in size from those of N16961. Thirty-seven of the amplicons exhibited differences in size. The diversity of the nontoxigenic O139 strain was found to be the most extensive in all the tested strains.

### Genome content and gene arrangement of the classical strains

In the classical strains tested, genome islands VPI-I, VPI-II and the O antigen synthesis gene cluster were detected, but VSP-I and VSP-II were not. However, the super integron region of the three toxigenic classical strains produced 29 PCR products (amplified using 16 pairs of primers) from 57 PCRs that were different from those obtained with N16961.

### RFLP analysis of amplicons with the same length in WGPScanning

One limitation of WGPScanning is that the sequence variations are unknown when the amplicons from different strains are identical in length. To investigate whether sequence variations existed in such amplicons, five strains, including the toxigenic El Tor strain N16961, classical strain 569B, toxigenic O139 strain MO45, and nontoxigenic El Tor strains 19–22 and JS32, were selected for RFLP analysis.

**Figure 3:**
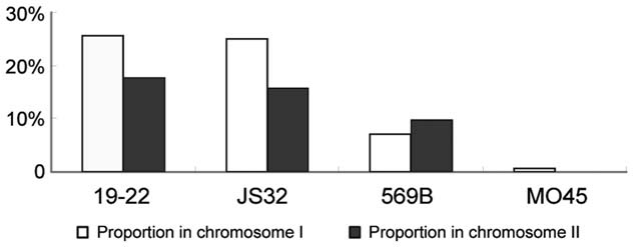
RFLP analysis of same-sized amplicons obtained using the same primers in the tested strains.

Each of the identical amplicons (in the selected five strains) from chromosomes I (208) and II (51) were subjected to RFLP analysis. Endonucleases that could digest a particular amplicon into fragments that could be easily separated by gel electrophoresis for comparison between strains were selected. One hundred forty (67.3%) and 38 (74.5%) segments from chromosomes I and II, respectively, exhibited digestion patterns identical to N16961. The highest, second highest and lowest differences ([number of segments yielding different digestion pattern]/[number of segments with identical amplicons]) were observed in the nontoxigenic El Tor strains, classical strain 569B and toxigenic O139 strain MO45, respectively (Figure 3).

### Detection of *rrn*-mediated rearrangements of large DNA fragments in toxigenic *V. cholerae* El Tor and O139

**Figure 4:**
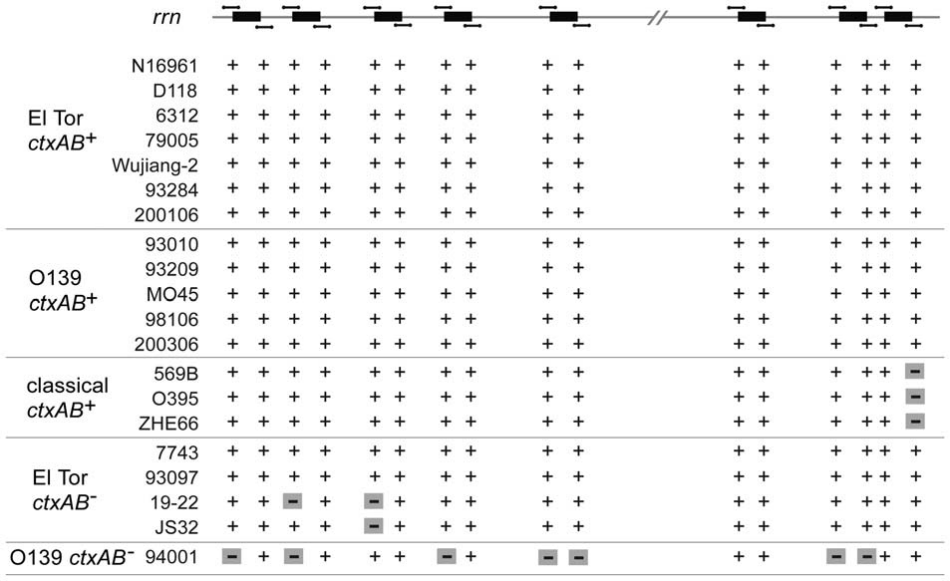
Schematic diagram of the *rrn* operons position in the test strains using PCR. In the top of the figure, the straight line indicated Chromosome I of N16961. The black rectangles represented the *rrn* operons in N16961 (not to scale). The upper and lower short lines represented the corresponding amplicons that were got with the primer pairs in the *rrn* operons and in the up and down adjacent sequence of *rrn* operons respectively. In the lower part of the figure in the PCR result matrix, “+” represented the amplicon was the same to that of N16961 while “−” represented no amplicon was got with the corresponding primer pairs in the tested strains.

The *rrn*-mediated rearrangement represents an important form of variation that occurs in bacteria. To determine whether such variation events have occurred in the tested strains, we analyzed the *rrn* operons and the immediately adjacent sequences by PCR and PFGE with I-*Ceu*I digestion. It was demonstrated that an I-*Ceu*I cutting site was present in each rRNA gene and at no other site in Gram-negative bacteria [15]. The number of *rrn* operons could be determined by analyzing the PFGE pattern observed after I-*Ceu*I digestion of the genome [19], [20]. Totally, eight I-*Ceu*I restriction sites were found exclusively in the *rrn* operons on Chromosome I of N16961 after analyzing the sequence of N16961 (data not shown). The I-*Ceu*I digestion profiles of the tested toxigenic El Tor strains could be divided into two groups. There was only one band difference between these two groups (data not shown). All of the five tested toxigenic O139 strains yielded identical I-*Ceu*I digested PFGE pattern. The relative positions of the *rrn* operons were detected by PCR, to amplify the each pair of junction regions of eight *rrn* operons and chromosome sequences. The PCR results of all tested toxigenic El Tor and O139 strains were identical to those in N16961 (Figure 4).

It has been reported that the fragment between the eighth and second *rrn* operons in N16961 was inverted in O395, which may have been the result of homologous recombination between *rrn* operons [21]. WGPScanning indicated that the forward and reverse primers located in the eighth *rrn* operon and the VC2760 gene of N16961 respectively (spanning 2,936,996– 2,946,013 nt) were unable to produce an amplicon in any of the three classical strains. In contrast, amplicons identical in size to those from N16961 were obtained from all other tested strains with that same pair of primers. The relative positions of *rrn* operons were amplified using primers designed based on the *rrn* operon and adjacent sequences in N16961. The results were the same in classical strains. Furthermore, PFGE patterns after I-*Ceu*I digestion were the same for the three classical strains.

PFGE patterns with I-*Ceu*I digestion were different between the four nontoxigenic El Tor strains. There was only one band difference (slightly in size of the bands) between the TCP^+^ nontoxigenic strains 93097 and 7743. However, five bands difference were observed between the two TCP^−^ nontoxigenic strains JS32 and 19–22. Relative positions detection of the *rrn* operons in TCP^+^ nontoxigenic strains were identical to those of N16961; however, amplification differences were observed in the second and third *rrn* operons between the TCP^−^ nontoxigenic strains and N16961 (Figure 4). We speculate that major sequence variation may have occurred in those regions.

### Genomic integration of the CTX prophage in toxigenic O1 and O139 strains

The El Tor strain N16961 was found to harbor a single El Tor-derived CTX prophage on chromosome I [22]. Different types and copy numbers of CTX prophages, which have integrated into chromosomes I and/or II, have been identified in toxigenic strains of different biotypes and serogroups [21], [23], [24]. In the first round of our WGPScanning, amplification diversity was observed in the CTX regions. To obtain more detailed information about the integration of CTX prophage into the toxigenic strains, PCR analyses were performed based on the reported integration site sequence [25]. The results indicated that there was diversity in both the copy number and site of integration for the CTX prophage in the tested O1 and O139 strains.

#### (i) CTX phage integration in the toxigenic El Tor strains

In the six tested toxigenic El Tor strains, two distinct amplification patterns were observed. Four of the strains (D118, 6312, Wujiang-2 and 79005) showed an identical amplification pattern. Amplicons of approximately 1 kb in size were got with primers designed to detect the CTX prophage integration in chromosome II, which was the same as that obtained in N16961. No CTX phage integrated into chromosome II in N 16961, amplification with primers located in the up and down stream of the integration site yielded a 1 kb amplicon in this strain. We hypothesized that CTX prophage had integrated only into chromosome I. In the remaining two strains (93284 and 200106), an amplicon of approximately 8 kb was got using primers designed to detect the CTX prophages integration in chromosome II, which indicated a CTX prophage integrated into chromosome II in these two strains. Amplicons of approximately 8 kb were got using primers located in VC1465 and *zot* respectively in both strain 93284 and 200106. This indicated that at least one CTX prophage also integrated into chromosome I in these two strains.

#### (ii) CTX phage integration in the toxigenic O139 strains

In three (93010, 93209 and MO45) of the five O139 strains, only amplicons of approximately 1 kb were obtained by using primers to the CTX prophage integration site in chromosome II, indicating that the CTX prophage only integrated in chromosome I. For the remaining two strains (98106 and 200306), the PCR analyses indicated that the CTX prophage had integrated into both of the chromosomes. However, amplicons of different size were got for chromosome I from these two strains, suggesting that different combination of CTX prophage integration and/or arrangements of the RS region and CTX core region occurred at the integration site.

#### (iii) The toxigenic O1 classical strains

Similar to the toxigenic El Tor and O139 strains, each of the three classical strains exhibited diversity in the genomic integration of the CTX prophage. The amplification results indicated that the CTX prophage existed in both of the chromosomes; however, the sizes of amplicons were distinct among these three strains, which indicated that copy number or gene organization of CTX prophage might differ in these strains.

## Discussion

In this study, we analyzed the genomic structures of the *V. cholerae* serogroups O1 and O139 using both WGPScanning and CGH approaches. We were not only able to detect gene deletions but also able to detect potential fragment insertion events among *V. cholerae* strains that have a highly similar genetic background.

### The syntenic genome structure of toxigenic O1 El Tor and O139 strains

CGH analysis of *V. cholerae* suggested that the toxigenic El Tor and O139 strains have conservative genome contents [17]. WGPScanning is able to provide useful information regarding fragment arrangement and insertion events. The toxigenic El Tor and O139 strains used in this study were isolated from different regions during different cholera epidemics that occurred over 40 years (1961 to 2001). In most regions of the genome, the results from the two analyses were consistent, which suggested that both the genome content and the gene arrangement were conservative among these strains. In addition, the findings from PFGE with I-*Ceu*I digestion and WGPScanning suggested that large DNA fragment rearrangements mediated by *rrn* operon recombination were actually relatively rare events in the tested toxigenic El Tor and O139 strains. Therefore, we concluded that the genomic structures of toxigenic El Tor and O139 *V. cholerae* were syntenic.

In this study, some regions yielded inconsistent results between the CGH and WGPScanning; therefore, the primers were redesigned to perform PCR detection again. In most cases, the results of the second round of PCR were consistent with those of CGH, suggesting that the minor nucleotide variation existed in these strains. The RFLP analysis of the identical amplicons amplified by the same primers in different strains also suggested that dispersed minor nucleotide variation may exist in the tested toxigenic strains. However, in some chromosomal regions these two methods gave inconsistent results, even after redesigning the primers used for WGPScanning. For those regions, we concluded that fragment insertions were likely to have occurred. The principal mechanism of genomic variation among these toxigenic strains thus appeared to be the random distributed minor nucleotide variation in conjunction with gene deletion and fragment insertion. Some molecular typing approaches, including RFLP, ribotyping, PFGE and AFLP analyses, have demonstrated the genomic diversity of toxigenic O1 and O139 strains [26], [27], [28], [29], [30], [31], [32]. Minor nucleotide variation may lead to the subtype differences. Genomic comparison of three *V. cholerae* whole genome sequences has revealed that SNPs represent a major type of variation in these bacteria [21]. This may partly explain the PCR-RFLP results of the identical amplicons.

### The subdivided lineages of TCP^+^ nontoxigenic O1 El Tor strains

The nontoxigenic O1 and O139 strains exhibited more gene deletions and greater sequence variation when compared to the toxigenic strains. However, higher genome similarities among the TCP^+^ nontoxigenic O1 El Tor strains (7743 and 93097) were observed in CGH and WGPScanning. This finding may suggest that acquisition of VPI was limited to certain strains with specific genome structures. These TCP^+^ nontoxigenic El Tor strains could be further classified into different groups, even though each possessed the O1 antigen gene cluster. Strain carrying El Tor type *tcpA* (7743) exhibited greater genomic similarity than did the strains carrying non-El Tor type *tcpA* (93097) and was readily infected by El Tor type CTXΦ and converted into a toxigenic strain [33]. Moreover, several variants of TCP exist, and they can mediate the infection of different types of CTXΦ to certain nontoxigenic strains at varying efficacies [33]. Several other nontoxigenic strains carrying the non-El Tor type *tcpA* were not as easily infected by the El Tor type CTXΦ, which was presumed to be the result of different pIII^CTX^ proteins recognizing specific TcpA as the receptor for phage infection [34].

TCP is the critical element in acquisition of the toxin gene through CTXΦ lysogenic conversion and contributes to the generation of a new toxigenic strain. A study has reported that the ribotype of a TCP^+^ nontoxigenic non-O1/non-O139 strain was quite similar to that of a toxigenic O1 strain, and the strain may represent an intermediate in the evolution towards pathogenic status [8]. We hypothesize that subdivided lineages, based on different TCP and genome structure, may exist in the TCP^+^ nontoxigenic O1 strains, which are believed to be intermediates of the toxigenic strains. Whether or not these strains can eventually evolve into toxigenic types not only depends on the environmental condition and interaction with CTXΦ, but also on the characteristics of the genomic structure, such as the possession of TCP. The seventh pandemic strains might derive from a lineage of nontoxignic strains carrying the El Tor type TCP, whereas the other TCP^+^ El Tor strains may become toxigenic via infection and conversion of other CTXΦ alleles. These toxigenic strains would be expected to form new predominant clones in the event that a new epidemic is established.

## Materials and Methods

### Strains

Thirty toxigenic and nontoxigenic O1/O139 strains were analyzed by CGH test in our previous study [18]. In the WGPScanning, 20 strains were selected from them, which covered all the serogroups, serotypes, biotypes, and *tcpA* sequence types. The details of the 20 experimental toxigenic and nontoxigenic O1 and O139 *V. cholerae* strains are shown in Table 1.

**Table 1:** Strains used for comparative analysis in this study.

Strain	Serogroup, biotype	Serotype	Year isolated	Geographic region	Source	*ctxAB*	*tcpA*
N16961	O1, El Tor	Inaba	1975	Bangladesh	Patient	+	+, El Tor type
D118	O1, El Tor	Ogawa	1961	China	Patient	+	+, El Tor type
6312	O1, El Tor	Ogawa	1961	Indonesia	Patient	+	+, El Tor type
79005	O1, El Tor	Ogawa	1979	China	Patient	+	+, El Tor type
Wujiang-2	O1, El Tor	Inaba	1980	China	Patient	+	+, El Tor type
93284	O1, El Tor	Ogawa	1993	China	Patient	+	+, El Tor type
200106	O1, El Tor	Inaba	2001	China	Water	+	+, El Tor type
93010	O139	N/A	1993	China	Patient	+	+, El Tor type
93209	O139	N/A	1993	China	Carrier	+	+, El Tor type
MO45	O139	N/A	1993	India	Patient	+	+, El Tor type
98106	O139	N/A	1998	China	Patient	+	+, El Tor type
200306	O139	N/A	2003	China	Patient	+	+, El Tor type
569B	O1, classical	Inaba	1948	India	Patient	+	+, classical type
O395	O1, classical	Ogawa	1964	India	Patient	+	+, classical type
ZHE66	O1, classical	Ogawa	1963	China	Carrier	+	+, classical type
7743	O1, El Tor	Ogawa	1977	China	Water	_	+, El Tor type
93097	O1, El Tor	Ogawa	1993	China	Patient	_	+, non El Tor type
19–22	O1, El Tor	Ogawa	1977	China	Patient	_	_
JS32	O1, El Tor	Inaba	1990	China	Patient	_	_
94001	O139	N/A	1994	China	Water	_	_

N/A, Not applicable.

**Table 2:** WGPScanning results for the tested strains.

Strain	Serogroup, biotype	Serotype	*ctxAB*	*tcpA*	No. of un-amplifiable	No. of amplicons smaller than N16961	No. of amplicons larger than N16961	No. of amplicons with same size as N16961	Proportion of different PCR products	Proportion of successful amplifications
D118	O1, El Tor	Ogawa	+	+, El Tor type	9	3	1	559	2.3%	98.4%
6312	O1, El Tor	Ogawa	+	+, El Tor type	9	2	0	561	1.9%	98.4%
79005	O1, El Tor	Ogawa	+	+, El Tor type	12	2	2	556	2.8%	97.9%
Wujiang-2	O1, El Tor	Inaba	+	+, El Tor type	5	3	7	557	2.6%	99.1%
93284	O1, El Tor	Ogawa	+	+, El Tor type	5	2	1	564	1.4%	99.1%
200106	O1, El Tor	Inaba	+	+, El Tor type	6	3	2	561	1.9%	99.0%
93010	O139	N/A	+	+, El Tor type	24	3	1	544	4.9%	95.8%
93209	O139	N/A	+	+, El Tor type	23	0	1	543	4.2%	95.1%
MO45	O139	N/A	+	+, El Tor type	29	2	3	538	5.9%	94.9%
98106	O139	N/A	+	+, El Tor type	14	4	4	550	3.8%	97.6%
200306	O139	N/A	+	+, El Tor type	16	4	3	549	4.0%	97.2%
569B	O1, classical	Inaba	+	+, classical type	52	6	4	510	10.8%	90.9%
O395	O1, classical	Ogawa	+	+, classical type	44	3	3	522	8.7%	92.3%
ZHE66	O1, classical	Ogawa	+	+, classical type	43	4	2	523	8.6%	92.5%
7743	O1, El Tor	Ogawa	_	+, El Tor type	27	0	1	544	4.9%	95.3%
93097	O1, El Tor	Ogawa	_	+, non El Tor type	50	2	11	509	11.0%	91.3%
19–22	O1, El Tor	Ogawa	_	_	92	14	12	454	20.6%	83.9%
JS32	O1, El Tor	Inaba	_	_	66	10	13	483	15.6%	88.5%
94001	O139	N/A	_	_	99	19	18	436	23.8%	82.7%

### Preparation of chromosomal DNA

All *V. cholerae* strains were grown in 5 ml of Luria-Bertani broth to an optical density (OD) of 0.8 at 600 nm. Chromosomal DNA was prepared using the NucleoSpin Tissue kit (Macherey-Nagel, Germany) following the manufacturer's protocol.

### Primer design

Based on the published genome sequence of O1 El Tor strain N16961 [22], 572 pairs of primers were designed using Oligo6.0 primer analysis software (Molecular Biology Insights, USA). Primers ranged in nucleotide (nt) length from 18 to 22 (most being 22 nt), and the average expected size of the amplicons was 8,207 base pairs (bps). The expected maximum and minimum sizes of the amplicons were 14,994 bps and 3,098 bps, respectively. Overlaps between adjacent segments ranged from 4 to 8,228 bps, with the average size being 1,155 bps.

### WGPScanning

PCR reactions were performed on a MJ Research PTC-200 (Bio-Rad, USA) amplification system using long accurate (LA) PCR Taq (TaKaRa, China). N16961 was used as the positive control. The PCR was performed in a 20 µl reaction mixture. The PCR conditions used were initial denaturation at 95°C for 5 min; 30 cycles of denaturation at 94°C for 45 s, annealing at 55–58°C for 30 s and extension at 72°C for 5–10 min; and a single final extension at 72°C for 7 min. The PCR reactions were repeated for the negative amplification reaction to determine the reliability of non-amplifiable samples.

### PCR-restriction fragment length polymorphism (PCR-RFLP)

For amplicons that were identically amplified with the same primers in each of the different tested strains, PCR-RFLP analysis was performed. Based on the sequence of N16961, appropriate endonucleases were chosen to digest the amplicon into fragments that could be easily separated by electrophoresis. The PCR-RFLP patterns of each of the different strains were then compared.

### Pulsed-field gel electrophoresis (PFGE)

PFGE was carried out following the PulseNet One-day Standardized PFGE Protocol for *V. cholerae* with minor modifications [35]. The electrophoresis was run on a CHEF-DR III variable angle system (Bio-Rad) with the parameters of 4–20 s for 20 h. Images were captured using a Gel Doc 2000 system (Bio-Rad) and converted to TIFF files for computer analysis.

### Variation analysis of *rrn* operon junction sequences

Primers were designed based on the internal sequence of each *rrn* operon and its corresponding upstream and downstream flanking sequences, according to the eight *rrn* operons and their flanking sequences on El Tor strain N16961 genome. PCR was performed to determine whether sequence variations existed within the junction sequences of the *rrn* operon.

### Cladogram reconstruction based on WGPScanning and CGH data

After the WGPScanning analysis, the PCR reaction products from the tested strains whose sizes were similar to, larger than, or smaller than that of N16961 were assigned values of 1, 2 and 0.75, respectively. A reaction with no PCR product was assigned a 0. The data were processed using a practical extraction and reporting language procedure. Trees based on the unweighted-pair group method using arithmetic average linkages (UPGMA) were reconstructed with BioNumerics version 3.0 software (Applied Maths BVBA, Belgium). In our previous research, the genomes of 30 toxigenic and nontoxigenic *V. cholerae* were investigated by CGH [18]. A cladogram was reconstructed with BioNumerics using the CGH results of the 20 strains selected from those 30. In this study, the same 20 strains were analyzed by WGPScanning. BioNumerics was also used to calculate the correlation coefficient between cladograms based on data from both WGPScanning and CGH.

## Supporting Information

Figure S1 **Comparison of CGH and WGPScanning of the tested strains (Chromosome I).** In the figure, the uppermost line represents the genome of N16961, with the points denoting 30-bp lengths. The position and direction of the open-reading frames (ORFs) in N16961 are annotated. The names of ORFs with intervals of 15 ORFs are also annotated. The names of the tested strains are on the left side of the figure. Results from CGH using the N16961 microarray are shown in the upper parts and those from WGPScanning in the lower parts. Results from CGH are presented as follows: blue spaces indicate the genes that were detected (present), yellow spaces indicate genes that were not detected (absent), and blank spaces indicate unidentified genes. Results from the WGPScanning analysis are presented as follows: gray color denotes segments identical in size to N16961 segments. Size differences are indicated in blue (larger) and yellow (smaller), while genes that were not amplified are in red.
(TIF)

Figure S2 **Comparison of CGH and WGPScanning of the tested strains (Chromosome II).** In the figure, the uppermost line represents the genome of N16961, with the points denoting 30-bp lengths. The position and direction of the open-reading frames (ORFs) in N16961 are annotated. The names of ORFs with intervals of 15 ORFs are also annotated. The names of the tested strains are on the left side of the figure. Results from CGH using the N16961 microarray are shown in the upper parts and those from WGPScanning in the lower parts. Results from CGH are presented as follows: blue spaces indicate the genes that were detected (present), yellow spaces indicate genes that were not detected (absent), and blank spaces indicate unidentified genes. Results from the WGPScanning analysis are presented as follows: gray color denotes segments identical in size to N16961 segments. Size differences are indicated in blue (larger) and yellow (smaller), while genes that were not amplified are in red.
(TIF)
