# Supplementary figures and images for: Whole Genome PCR Scanning Reveals the Syntenic Genome Structure of Toxigenic Vibrio cholerae Strains in the O1/O139 Population
Source: PLoS One. 2011 Aug 31;6(8):e24267. doi: 10.1371/journal.pone.0024267 (PMC3164192; doi:10.1371/journal.pone.0024267)

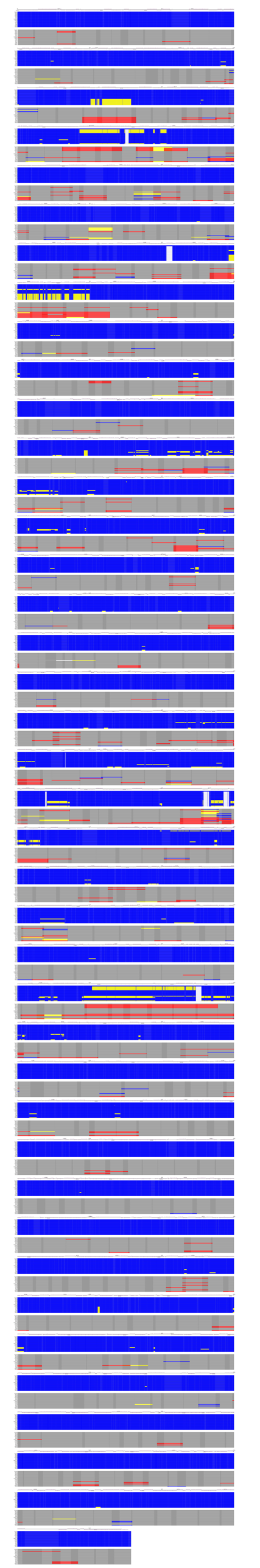

Supplement: Figure S1 — Comparison of CGH and WGPScanning of the tested strains (Chromosome I). In the figure, the uppermost line represents the genome of N16961, with the points denoting 30-bp lengths. The position and direction of the open-reading frames (ORFs) in N16961 are annotated. The names of ORFs with intervals of 15 ORFs are also annotated. The names of the tested strains are on the left side of the figure. Results from CGH using the N16961 microarray are shown in the upper parts and those from WGPScanning in the lower parts. Results from CGH are presented as follows: blue spaces indicate the genes that were detected (present), yellow spaces indicate genes that were not detected (absent), and blank spaces indicate unidentified genes. Results from the WGPScanning analysis are presented as follows: gray color denotes segments identical in size to N16961 segments. Size differences are indicated in blue (larger) and yellow (smaller), while genes that were not amplified are in red. (TIF) [file pone.0024267.s001.tif]

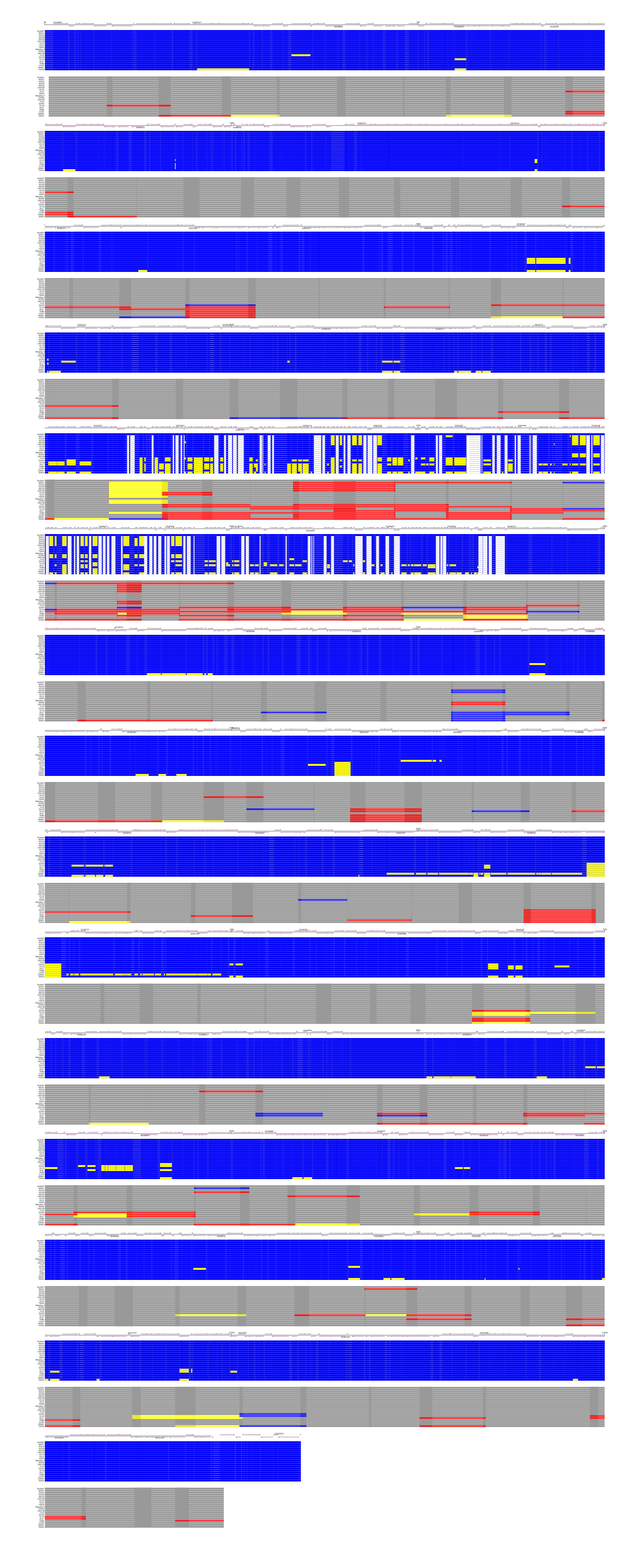

Supplement: Figure S2 — Comparison of CGH and WGPScanning of the tested strains (Chromosome II). In the figure, the uppermost line represents the genome of N16961, with the points denoting 30-bp lengths. The position and direction of the open-reading frames (ORFs) in N16961 are annotated. The names of ORFs with intervals of 15 ORFs are also annotated. The names of the tested strains are on the left side of the figure. Results from CGH using the N16961 microarray are shown in the upper parts and those from WGPScanning in the lower parts. Results from CGH are presented as follows: blue spaces indicate the genes that were detected (present), yellow spaces indicate genes that were not detected (absent), and blank spaces indicate unidentified genes. Results from the WGPScanning analysis are presented as follows: gray color denotes segments identical in size to N16961 segments. Size differences are indicated in blue (larger) and yellow (smaller), while genes that were not amplified are in red. (TIF) [file pone.0024267.s002.tif]
